# Induction of antitumor response to fibrosarcoma by Newcastle disease virus-infected tumor vaccine

**DOI:** 10.1007/s12032-017-1034-y

**Published:** 2017-09-01

**Authors:** Mai Takamura-Ishii, Takahiro Miura, Takaaki Nakaya, Katsuro Hagiwara

**Affiliations:** 1 School of Veterinary Medicine, Rakuno Gakuen University, 582 Midorimachi, Bunkyodai, Ebetsu, Hokkaido 069-8501 Japan; 2 Department of Infectious Diseases, Kyoto Prefectural University of Medicine, 465 Kawaramachi-hirokoji, Kamigyo-ku, Kyoto, Japan

**Keywords:** Fibrosarcoma, Immunotherapy, Newcastle disease virus, Tumor vaccine

## Abstract

Fibrosarcoma is a locally aggressive malignant tumor with a high recurrence rate, so that wide excisional surgery is necessary for treatment. However, it is often difficult to resect with a sufficient margin of excision at the site of tumor infiltration. Recombinant tumor vaccine therapy is a useful method to induce specific immunity. In this study, we have shown its utility as a candidate for therapy by applying a recombinant Newcastle disease virus (rNDV) tumor vaccine (rNDV-TV). Although the therapeutic effect of similar viruses has been examined in several tumors, the vaccination efficacy against fibrosarcoma has not been demonstrated until now. In this study, we showed the induction of an antitumor response by rNDV-TV against murine fibrosarcoma and investigated the role of lymphocytes in tumor elimination. Intraperitoneal inoculation of murine fibrosarcoma (WEHI164) cells showed increased lethality in C.B.17*scid/scid* (scid) mice within 2 weeks of inoculation. The survival rate increased to 80% when the mice were transfused with CD3^+^ cells from BALB/c mice previously immunized with rNDV-TV. However, all mice died from tumor growth after inoculation with non-immunized CD3^+^ cells. Although the survival rate was around 50% in mice receiving only immunized CD4^+^ and CD8^+^ cells, the survival rate was not decreased in mice receiving CD3^+^CD4^−^CD8^−^ (natural killer T; NKT) cells together with immunized CD4^+^ and CD8^+^ cells. This study showed rNDV-TV induced an antitumor T cell response to WEHI164 cells, and major subsets of cells involved in tumor exclusion were CD4^+^ and CD8^+^ cells, together with NKT cells.

## Introduction

Soft tissue sarcomas are rare neoplasms accounting for around 1% of all tumors [1]. Fibrosarcoma is one of more than 50 histologic subtypes of soft tissue sarcoma, as classified by the World Health Organization (WHO). In a recent evaluation, it was shown that 50% of patients with fibrosarcoma died of locally aggressive and/or metastatic disease (median follow-up 1 year; range <1–8 years) [2]. Although local control after surgery is important for reducing the high rate of local recurrence [3], radiotherapy is not sufficiently effective at present [4], and the prognosis of patients with soft tissue sarcoma has not improved during the last 20 years [5]. These facts indicate that currently used treatments are not effective for tumor elimination; therefore, alternative treatments for the prevention of postoperative recurrence are required. Currently, immunotherapy is being investigated as a fourth therapeutic option, in addition to surgical therapy, radiotherapy, and chemotherapy, for the treatment of tumors. One such immunotherapeutic approach employs oncolytic viruses to target tumors.

Newcastle disease virus (NDV) is a negative-sense, single-stranded RNA virus that can infect tumor cells and has oncolytic properties [6]. NDV-infected tumor cells produce type I interferons (IFNs) and induce expression of major histocompatibility complex (MHC) class I and cell adhesion molecules, such as hemagglutinin-neuraminidase (HN) protein, on the tumor cell surface, increasing interaction between tumor cells and T cells [7]. Additionally, NDV-infected tumor cells induce interleukin (IL)-2 production and proliferation of helper T (Th) cells and prevent T cell anergy by costimulation via CD28 molecules [8]. NDV is considered to be a promising adjuvant for tumor vaccines, because type I IFNs produce antiviral effects not only by activating innate immunity, but also by activating acquired immunity via induction of dendritic cell maturation [9, 10]. Presently, an NDV-infected tumor vaccine (NDV-TV), incorporating the avirulent Ulster strain of NDV as an adjuvant, is showing good results in several clinical trials. With head and neck squamous cell carcinoma, 5-year overall survival of vaccinated patients at stage 3 and 4 was significantly improved; moreover, delayed-type hypersensitivity (DTH) reactions were significantly increased in vaccinated patients compared with control patients [11]. Vaccinated patients with glioblastoma multiforme showed significantly prolonged median progression-free survival and median overall survival compared with control patients [12]. Extension of survival time was also recognized in breast cancer and colorectal cancer; however, the effect of NDV-TV on fibrosarcoma has not yet been examined [13, 14]. Previously, we generated a green fluorescent protein (GFP)-expressing recombinant NDV (rNDV). The virus induced cell death only in infected cells without producing any infectious virus particles; this was achieved by transforming the viral F protein cleavage site amino acid sequence to an attenuated virus type (G-R-Q-G/S-R↓L) [15]. Although NDV is known not to induce severe side effects in mammals [16], rNDV is safer than wild-type NDV for the inoculation of patients. Therefore, in this study, we examined antitumor effects induced by rNDV-TV in murine fibrosarcoma and a lymphocyte subset involved in tumor exclusion.

## Materials and methods

### Animals

Specific pathogen-free 8-week-old male and female BALB/c mice and 10-week-old C.B.17scid/scid (scid) mice were obtained from CLEA Japan, Inc. (Tokyo, Japan). Mice were maintained in a sterile isolator and were treated according to the Laboratory Animal Control Guidelines at Rakuno Gakuen university (approval number: VH14A4).

### Cell culture and virus production

The murine fibrosarcoma cell line WEHI164, derived from BALB/c mice, was used. Cells were cultured in RPMI-1640 medium (Sigma, USA), supplemented with 5% fetal calf serum (FCS; Biological Industries, USA), 200 U/mL penicillin, and 200 μg/mL streptomycin, at 37 °C in a humidified atmosphere with 5% CO_2_.

GFP-expressing rNDV was generated as previously described [15] and proliferated in embryonated chicken eggs. rNDV was collected from the allantoic fluid, and the number of focus-forming units (FFUs) was determined.

### Preparation of rNDV-infected WEHI164 cell vaccine (WEHI-NDV)

WEHI164 cells (10^5^ cells/well) were cultured with RPMI-1640 containing 5% FCS in 6-well plates. Then, the cells were infected with rNDV using a multiplicity of infection (MOI) of 2 and cultured at 37 °C for 24 h in a CO_2_ incubator. rNDV-infected tumor cells were harvested with ethylenediaminetetraacetic acid and phosphate-buffered saline (EDTA-PBS) and inactivated with UV irradiation (400 mJ/cm^2^). After washing with PBS, the cells were resuspended at 5 × 10^5^ cells/mL in RPMI-1640.

### Immunization protocol

Two vaccines and a control (WEHI-NDV, WEHI164 irradiated with UV (WEHI-UV), or RPMI-1640) were administered intraperitoneally to BALB/c mice at 10^5^ cells/mouse four times at weekly intervals.

### Separation of lymphocyte subsets from splenocytes

Spleen cells were collected from the mice, and splenic mononuclear cells (SMCs) were separated by density gradient centrifugation using Ficoll–Conray solution (*d* = 1.088). The separated SMCs were then conjugated with four kinds of monoclonal antibodies (against CD3, CD4, CD8, and CD49b) and separated using an autoMACS separator (Miltenyi Biotec, Germany) according to the manufacturer’s guidelines. Isolated CD3^+^, CD4^+^, CD8^+^, and CD49b^+^ cells were used for immune monitoring experiments.

### Cytotoxicity assay

SMCs from each group of mice were cocultured with UV-irradiated (400 mJ/cm^2^) WEHI164 cells for 5 days at an effector–target ratio (E:T) of 4:1. After 5-day prestimulation with the tumor cells, effector SMCs were separated by density gradient centrifugation with Ficoll–Conray solution. SMCs were washed with PBS and cocultured with WEHI164 at an E:T of 20:1 for 24 h, and cytotoxicity was determined by lactate dehydrogenase (LDH) release using a Cytotoxicity Detection Kit^PLUS^ (LDH) (Roche Diagnostics, Switzerland). The cytotoxicity rate was calculated as follows: Cytotoxicity (%) = (Experimental LDH − Effector spontaneous LDH − Target spontaneous LDH)/(Target maximum LDH − Target spontaneous LDH) × 100.

### Transfusion of lymphocytes and tumor inoculation

Separated lymphocytes (CD3^+^, CD4^+^, CD8^+^, and CD49b^+^) were suspended in RPMI 1640, and 3–4 × 10^7^ cells/mouse (0.2 mL) were intraperitoneally administered in various combinations into scid mice. Two days after lymphocyte inoculation, 5 × 10^5^ WEHI164 cells/mouse were intraperitoneally administered, and all mice were observed for 4 weeks. After the observation period, all mice were euthanized and a pathological investigation was performed. Mice that died during the observation period were subject to the same procedure.

### Flow cytometry

To monitor lymphocyte subsets in the peripheral blood mononuclear cells (PBMCs) from each group of mice, the separated cells were incubated with rat anti-mouse CD4-FITC mAb and rat anti-mouse CD8a/Lyt-2-PE, or hamster anti-mouse CD3ε-PE (all Beckman Coulter, USA) and FITC-conjugated rat anti-mouse CD49b/pan-NK cells (BD Pharmingen™, USA) for 30 min at room temperature. The cells were then washed with PBS twice, treated with 0.5% formalin-PBS, and used for flow cytometry analysis (Beckman Coulter, USA).

### Cytokine gene expression

To monitor cytokine gene expression after tumor stimulation, the separated splenocytes from immunized and non-immunized mice were used as effector cells and WEHI164 cells were used as target cells. Effector cells and target cells were cocultured at an E:T of 20:1 for 6 h at 37 °C. After coculture, the cells were treated with TRIzol (Invitrogen, USA), and RNA was extracted following a standard method. Briefly, the cells were completely lysed with 0.5 mL of TRIzol reagent, and 0.1 mL of chloroform was added. After incubation for 5 min at room temperature, the samples were centrifuged for 10 min at 16,440×*g* at 4 °C, and a colorless upper aqueous phase containing the RNA was transferred to a new tube. Then, 0.25 mL of isopropanol was added to the aqueous phase, and the samples were incubated for 10 min. The samples were centrifuged for 10 min at 16,440×*g* at 4 °C, and the supernatant was discarded. The RNA pellet was resuspended in 1 mL of 75% ethanol, and the samples were centrifuged for 10 min at 16,440×*g* at 4 °C. The supernatant was discarded, and then the pellet was dried and resuspended in 25 µL of RNase-free water. For cDNA synthesis, RNA was reverse-transcribed using a Transcriptor First Strand cDNA Synthesis Kit (Roche Diagnostics, Switzerland). Briefly, 1 μg of total RNA was reverse-transcribed using oligo(dT) primers and incubated for 60 min at 50 °C after denaturation. The reverse transcriptase was then inactivated by heating to 85 °C for 5 min.

### Quantitative polymerase chain reaction (qPCR)

qPCR was carried out using a LightCycler 2.0 (Roche Diagnostics, Switzerland) and the genes were detected with a QuantiTect SYBR Green Kit (Qiagen, Germany). The amplification conditions consisted of 45 cycles of 94 °C for 15 s, 60 °C for 30 s, and 72 °C for 15 s. The following primers were used: IFN-γ forward primer, 5′-TGAAAGCCTAGAAAGTCTGAATAAC-3′ and reverse primer, 5′-GTTGTTGCTGATGGCCTGAT-3′; GAPDH forward primer, 5′-CGTGAGTGGAGTCATACTGGAA-3′ and reverse primer, 5′-AACGGATTTGGCCGTATTG-3′. The expression of IFN-γ was normalized to that of the housekeeping gene, GAPDH.

### Statistical analyses

Differences between two groups of data were calculated with the Student’s *t* test. Statistical significance of the cytotoxicity assay was evaluated using the Tukey–Kramer multiple comparison method. Correlation analyses were performed using Spearman’s correlation coefficient. Values were regarded as significant at *p* < 0.05. The Student’s t test was performed with Excel software, and all other tests were calculated with R version 3.2.5.

## Results

### Antitumor response in BALB/c mice vaccinated with WEHI-NDV

**Fig. 1:**
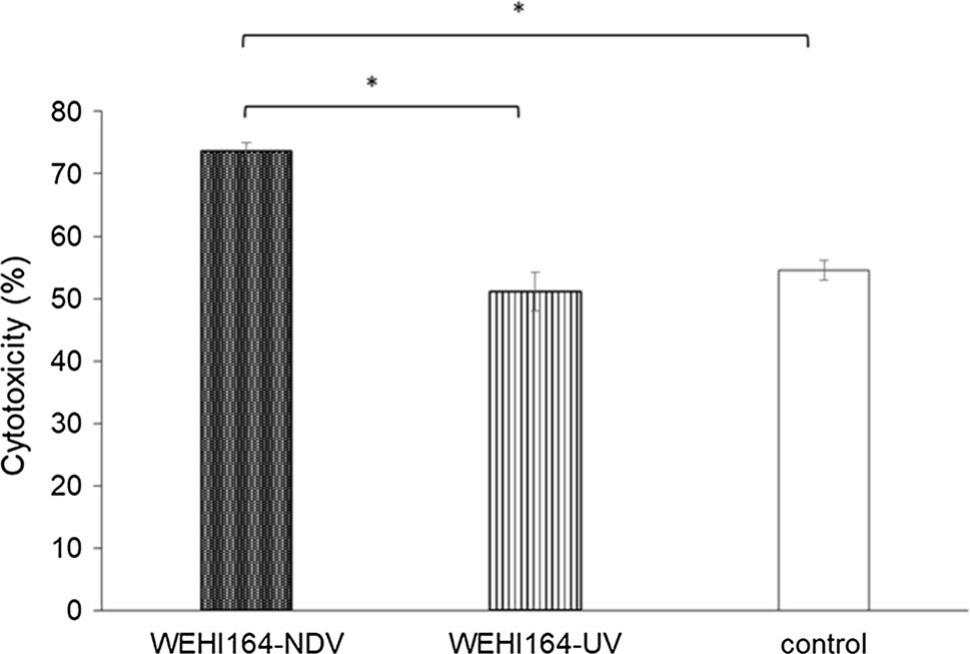
Induction of antitumor response by Newcastle disease virus (NDV)-infected WEHI164 cell vaccines (WEHI-NDV). WEHI-NDV, WEHI-UV (WEHI subjected to ultraviolet irradiation), or RPMI 1640 medium were administered to BALB/c mice, and splenocytes were cocultured with UV-irradiated WEHI164 cells for 5 days, 1 week after the last vaccination. After coculture of the collected splenocyte mononuclear cells (SMCs) with WEHI164 for 24 h, cytotoxicity was measured by quantifying lactate dehydrogenase (LDH) in the supernatant (* *p* < 0.05)

To confirm the antitumor response in mice vaccinated with WEHI-NDV, WEHI-UV, or RPMI 1640 medium, tumor cell cytotoxicity levels were investigated. The average cytotoxicity to WEHI164 cells was 73.6% in the mice immunized with WEHI-NDV. This was significantly higher than that of the WEHI-UV group (*p* = 0.01) or the control group (*p* = 0.02). These results suggested that WEHI-NDV vaccination increased the antitumor response to WEHI164 cells in BALB/c mice (Fig. 1).

### Survival rates in tumor-bearing scid mice

**Fig. 2:**
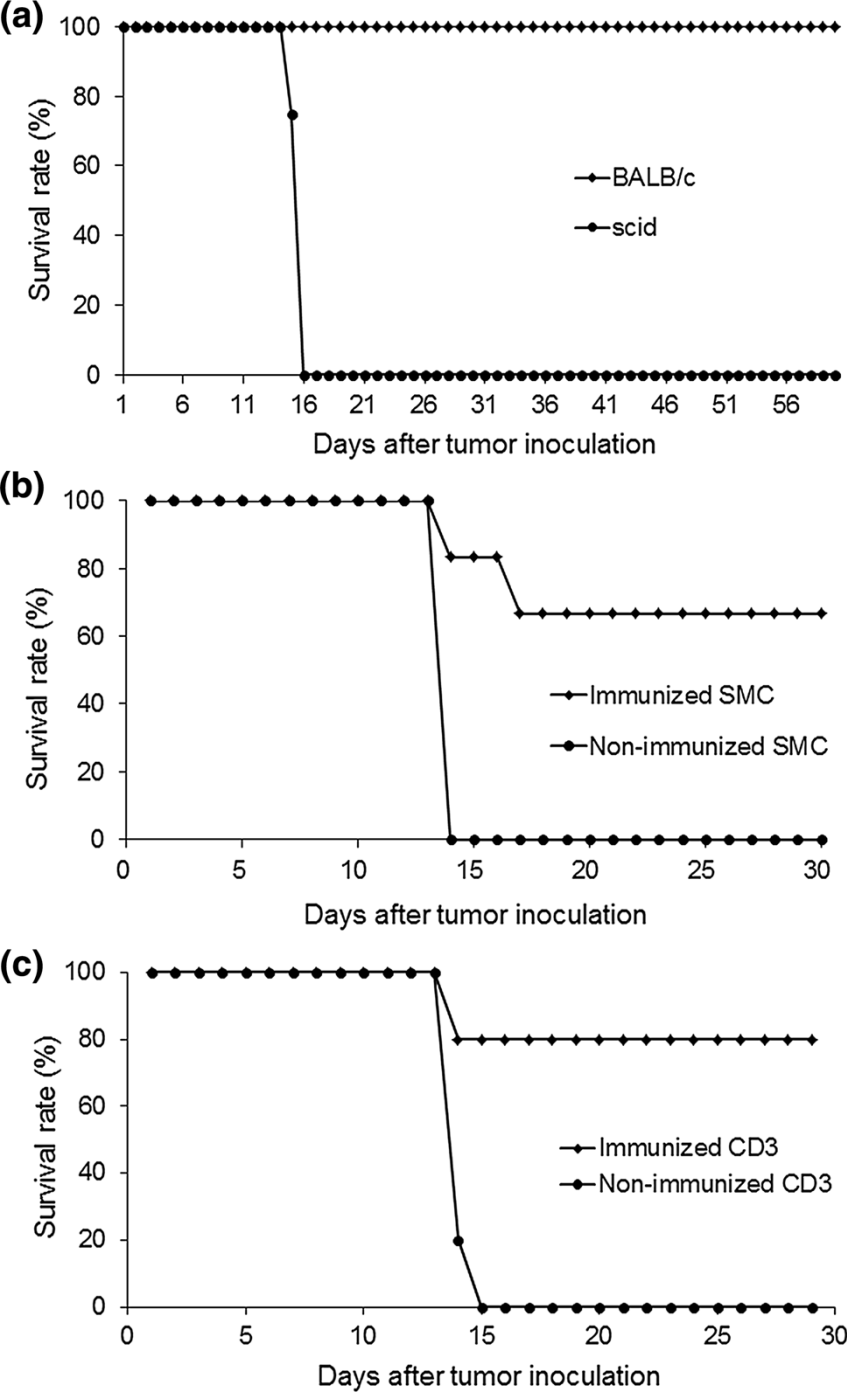
**a** Survival rates after WEHI164 inoculation in BALB/c and scid mice: WEHI164 were intraperitoneally administered to BALB/c and scid mice, and the mice were observed for 60 days. **b** Survival rate in splenocyte mononuclear cell (SMC)-transfused scid mice after WEHI164 cell inoculation: immunized or non-immunized SMCs were transfused. After tumor cell inoculation, the mice were observed for 30 days. **c** Survival rates in CD3^+^ cell-transfused scid mice after WEHI164 cell inoculation: immunized or non-immunized CD3^+^ cells were transfused. After tumor inoculation, the mice were observed for 4 weeks

To investigate the mortality of tumor-bearing mice, scid mice and BALB/c mice were inoculated with WEHI164 cells. There was no lethality in BALB/c mice for 60 days after tumor inoculation, and the tumors were completely excluded from the peritoneal cavity. On the contrary, all scid mice were dead within approximately 2 weeks (average 14.7 days) of tumor inoculation (Fig. 2a). Pathological examination showed extensive intraperitoneal tumor metastasis in the dead scid mice.

### Antitumor effects of SMC transfusion

To examine the antitumor effects of tumor antigen-sensitized T cells, SMCs from BALB/c mice that survived tumor inoculation were transfused into scid mice before tumor inoculation. The survival rate in the mice transfused with SMCs from surviving BALB/c mice was 60%, but all mice transfused with SMCs from non-treated BALB/c mice died; the mean survival was 18.3 days (data not shown).

WEHI-NDV or RPMI-1640 medium was administered to BALB/c mice. SMCs from these mice were transfused into scid mice, and WEHI164 cells were administered to the scid mice. As shown in Fig. 2b, the survival rate at 30 days after tumor inoculation was 71.4% in the immunized SMC transfusion group, whereas the survival rate in the non-immunized SMC transfusion group was 0% (mean survival was 12.0 days).

### Antitumor effects of CD3^+^ cell transfusion

To examine which cells types among SMCs have antitumor effects, purified CD3^+^ cells (immunized or non-immunized) were transfused into scid mice, and then WEHI164 cells were administered intraperitoneally. Of the mice that received immunized CD3^+^ cells, 80% survived at 4 weeks after tumor inoculation, whereas all control mice died; the mean survival was 14.2 days after non-immunized CD3^+^ cell transfusion (Fig. 2c). Pathological examination showed no intraperitoneal tumor growth in the mice that received immunized CD3^+^ cells; however, intraperitoneal dissemination of tumors with an accumulation of hemorrhagic ascites in the abdominal cavity was observed in the dead control mice.

### Antitumor effects of CD4^+^ and CD8^+^ cell transfusion

**Fig. 3:**
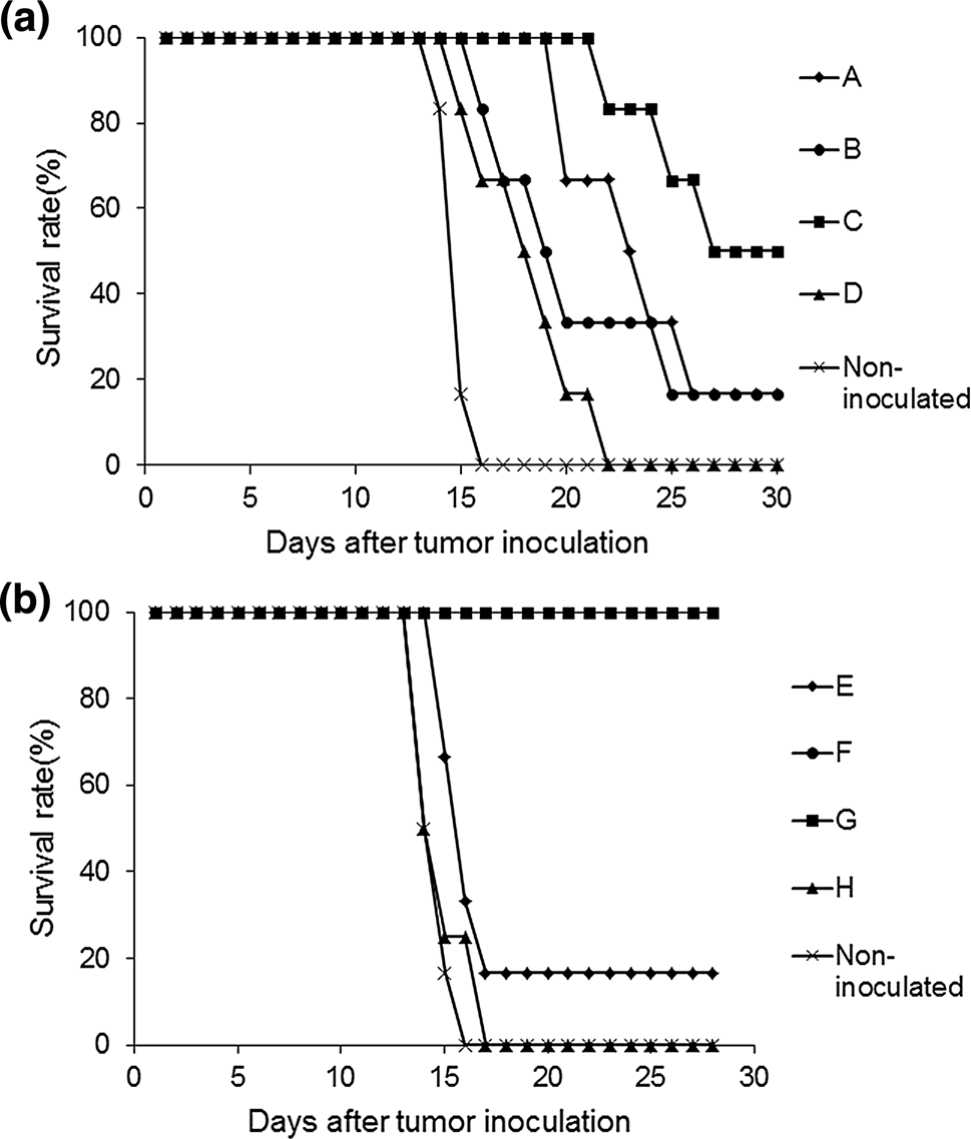
**a** Survival rate in CD4^+^ and CD8^+^ cell-transfused scid mice after WEHI164 cell inoculation: Immunized or non-immunized CD4^+^ and CD8^+^ cells were transfused into scid mice; after tumor inoculation, the mice were observed for 30 days. **b** Survival rate in CD49^+^ cell and CD3^+^CD49b^−^ cell-transfused scid mice after WEHI164 inoculation: Immunized or non-immunized CD49^+^ cells and CD3^+^CD49b^−^ cells were transfused into scid mice; after tumor inoculation, the mice were observed for 4 weeks

CD8^+^ cytotoxic T cells and CD4^+^ helper T cells are responsible for antigen-specific adaptive immune responses among CD3^+^ cells; therefore, we investigated which cells contributed to the antitumor effects. Immunized and/or non-immunized CD4^+^ cells and CD8^+^ cells were transfused to scid mice in 5 combinations (groups A to D and the non-inoculated group, as shown in Table 1). The survival rate was 16.7% in group A and B, 50% in group C, and 0% in the non-transfused group and group D at 4 weeks after tumor inoculation. The mean survival in the non-transfused group and group D was 15 and 18.3 days, respectively (Fig. 3a). There was no difference between the survival rate of groups A and B at 4 weeks after tumor inoculation. Although intraperitoneal tumors were observed in surviving individuals in groups A and B, no ascites was observed in group A. The surviving mouse in group C was different from other immunized CD3^+^ cell-transfused mice, in that it did not show complete tumor elimination.

**Table 1:** Groups of mice transfused with several combinations of lymphocytes

Group	Immunized				Non-immunized			
	CD4^+^	CD8^+^	CD49b^+^	CD3^+^CD49b^−^	CD4^+^	CD8^+^	CD49b^+^	CD3^+^CD49b^−^
A	+	–	–	–	–	–	–	–
B	–	+	–	–	+	–	–	–
C	+	+	–	–	–	–	–	–
D	–	–	–	–	+	+	–	–
E	–	–	+	–	–	–	–	+
F	–	–	–	+	–	–	+	–
G	–	–	+	+	–	–	–	–
H	–	–	–	–	–	–	+	+
Non-inoculated	–	–	–	–	–	–	–	–

Transfused cells are indicated with “+”

### Antitumor effects of CD49b^+^ and CD3^+^CD49b^−^ cell transfusion

**Fig. 4:**
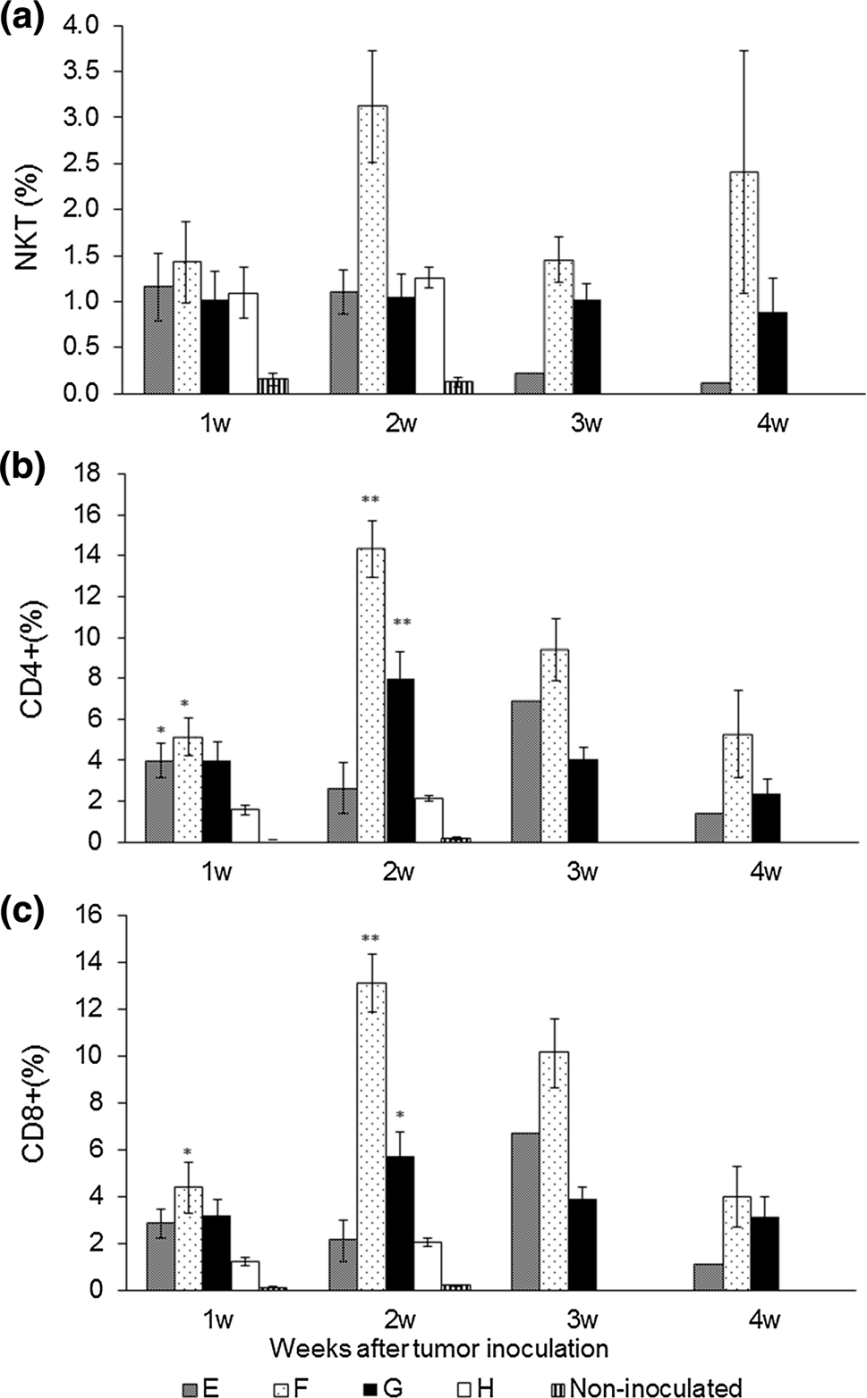
Subsets of CD49^+^ and CD3^+^CD49b^−^ lymphocytes transfused into scid mice after WEHI164 cell inoculation. After tumor inoculation, the ratio of peripheral **a** natural killer T (NKT) cells (CD3^+^CD49b^+^), **b** CD4^+^, and **c** CD8^+^ cells was measured each week by flow cytometry analysis (* *p* < 0.05, ** *p* < 0.01)

To confirm the antitumor response by CD49b^+^ cells (NK and NKT cells), isolated CD49b^+^ and CD3^+^49b^−^ cells (from immunized or non-immunized BALB/c mice) were transfused into scid mice. The transfused cells were combined according to Table 1, and the mice were divided into 5 groups (groups E to H and the non-inoculated group). The survival rate was 100% in groups F and G, 16.7% in group E, and 0% in the non-transfused group and group H at 4 weeks after tumor inoculation. The mean survival in the non-transfused group and group H was 15 and 14.7 days, respectively (Fig. 3b). There was no intraperitoneal tumor growth in mice transfused with immunized CD3^+^CD49b^−^ cells (both group F and G). In addition, there was no significant difference in the ratio of peripheral NKT cells among any groups when compared with group H (Fig. 4a). The ratio of peripheral CD4^+^ cells and CD8^+^ cells significantly increased at 2 weeks after tumor inoculation in group F (CD4^+^ (%), *p* = 0.0003; CD8^+^ (%), *p* = 0.0002) and group G (CD4^+^ (%), *p* = 0.007; CD8^+^ (%), *p* = 0.014) when compared with group H (Fig. 4b, c). Further, when Spearman’s correlation coefficient (*r*_s_) was calculated, there was significant positive correlation between number of survival days and the ratio of peripheral CD4^+^ cells (*r*_s_ = 0.662, *p* < 0.01) or CD8^+^ cells (*r*_s_ = 0.650, *p* < 0.01) at 2 weeks after tumor inoculation.

### Expression of IFN-γ mRNA in immunized splenocytes

**Fig. 5:**
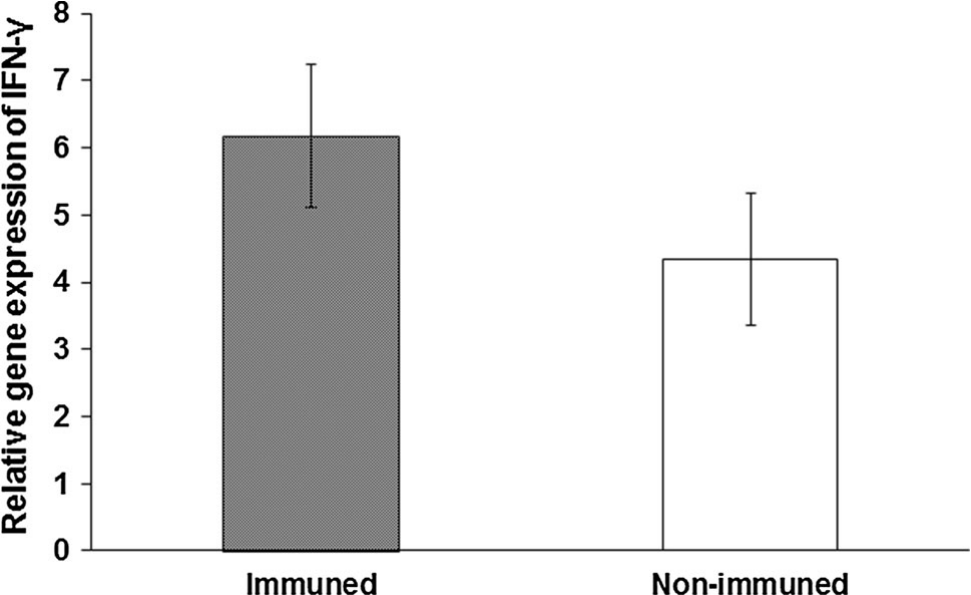
IFN-γ mRNA expression in immunized and non-immunized splenocytes cocultured with WEHI164 cells. IFN-γ mRNA expression in immunized and non-immunized splenocytes cocultured with WEHI164 was measured by quantitative PCR. mRNA expression was normalized to GAPDH mRNA expression level

In order to examine whether IFN-γ mRNA expression was induced in immunized splenocytes, gene expression was compared in immunized and non-immunized splenocytes cocultured with WEHI164 cells. Relative IFN-γ mRNA expression values were 6.17 in immunized splenocytes and 4.35 in non-immunized splenocytes at 6-h stimulation (Fig. 5). Although there was no significant difference between the groups (*p* = 0.277), immunized splenocytes showed relatively higher expression of IFN-γ than non-immunized cells.

## Discussion

In this study, we showed an effective antitumor response to WEHI164 cells induced by our rNDV-infected WEHI164 cell tumor vaccine. Tumor cells are known to have low immunogenicity, and this allows them to escape detection by the immune system. Therefore, in the development of tumor vaccines, it is essential to increase the immunogenicity of tumor cells using effective adjuvants to increase the immune response. NDV infects tumor cells specifically [17–19], and increases expression of MHC class I on the tumor cell surface and immune activity via induction of type I IFNs [7]. SMCs from BALB/c mice inoculated with WEHI-NDV, WEHI-UV, or culture medium were assessed for cytotoxicity to WEHI164 cells, and the WEHI-NDV group showed significantly higher cytotoxicity than the WEHI-UV and medium groups (Fig. 1). These results indicated that rNDV effectively induced an antitumor response when used as an adjuvant.

Although all BALB/c mice inoculated with WEHI164 cells survived and excluded the tumor cells, the scid mice (lacking T and B cells) showed tumor growth and died of peritoneally disseminated tumors within 2 weeks of tumor cell inoculation (Fig. 2a). These results showed that CD3^+^ cells were necessary for WEHI164 cell elimination.

Further, CD3^+^ cells derived from BALB/c mice immunized with WEHI-NDV or medium were transfused into scid mice before inoculation with WEHI164 cells. The mice that received non-immunized CD3^+^ cells were all dead within 15 days of tumor inoculation; on the other hand, the survival rate was 80% in the mice receiving immunized CD3^+^ cells, and tumor cells were excluded in all the survivors (Fig. 2c).

When immunized CD4^+^ cells and immunized CD8^+^ cells were transfused into scid mice, the survival rate was 50% at 4 weeks after tumor inoculation, but the tumors persisted intraperitoneally (Fig. 3a). Immunized CD4^+^ cells and non-immunized CD8^+^ cells transfused into scid mice extended survival, compared with the mice receiving non-immunized CD4^+^ and CD8^+^ cells, but the difference was not significant. These results indicated that transfusion of non-immunized lymphocytes did not induce the antitumor effect to extend survival. Meanwhile it was suggested T cells (CD4^+^ cells and CD8^+^ cells) from WEHI-NDV-immunized mice contributed to antitumor response induction, and immunized CD4^+^ cells were necessary to eliminate the tumors, leading to extended survival. As survival rates and tumor exclusion decreased in mice receiving immunized CD4^+^ and CD8^+^ cells, compared with those receiving immunized CD3^+^ cells, it was suggested that NKT cells, CD3^+^CD4^−^CD8^−^, might increase antitumor response induction by WEHI-NDV.

Next, CD49b^+^ cells, including NK and NKT cells, and CD3^+^CD49b^−^ cells, including T cells, were transfused into scid mice. All scid mice receiving immunized CD3^+^CD49b^−^ cells and immunized or non-immunized CD49b^+^ cells survived, and tumors were excluded. Further, in scid mice transfused with non-immunized CD3^+^CD49b^−^ cells and immunized or non-immunized CD49b^+^ cells, the survival rates were 0 and 16.7%, respectively, and tumors proliferated in the peritoneal cavity of surviving mice (Fig. 3b). These results showed that NK and NKT cells contributed to the antitumor response in the presence of immunized CD3^+^CD49b^−^ cells.

Although it is known that NKT cells play a role in the antitumor response [20], much remains unknown about the antitumor response of NKT cells, such as the recognition of tumor cells and their precise role in the antitumor effects. In one report, it was suggested that perforin derived from NKT cells was not essential for the antitumor response, but effector cells, such as NK cells and CD8^+^ cells, were activated via IFN-γ derived from NKT cells [21]. In this study, NKT cells could not exclude tumor cells in the absence of immunized T cells. Therefore, NKT cells might induce a strong antitumor response by activating immunized T cells and NK cells. Further, there was significant positive correlation between survival time and peripheral CD4^+^ cell ratio or CD8^+^ cell ratio at 2 weeks after tumor inoculation. These results confirmed the importance of CD4^+^ and CD8^+^ cells in tumor exclusion by rNDV-TV.

The results of this study show that rNDV-TV is a promising candidate for the treatment of fibrosarcoma. Further, it was suggested that the induction of the antitumor response by rNDV-TV involved NKT cells and NK cells cooperating with immunized T cells to exclude tumor cells. Our results are encouraging, and rNDV-TV may be a new strategy for the prevention of postoperative recurrence of fibrosarcoma. We have identified a possible method of survival prolongation with rare neoplasms.
